# Survivals of Angiography-Guided Percutaneous Coronary Intervention and Proportion of Intracoronary Imaging at Population Level: The Imaging Paradox

**DOI:** 10.3389/fcvm.2022.792837

**Published:** 2022-02-24

**Authors:** Andrew Kei-Yan Ng, Pauline Yeung Ng, April Ip, Lap-Tin Lam, Chung-Wah Siu

**Affiliations:** ^1^Cardiac Medical Unit, Grantham Hospital, Hong Kong, Hong Kong SAR, China; ^2^Department of Adult Intensive Care, Queen Mary Hospital, Pok Fu Lam, Hong Kong SAR, China; ^3^Division of Respiratory and Critical Care Medicine, Department of Medicine, Li Ka Shing Faculty of Medicine, The University of Hong Kong, Pok Fu Lam, Hong Kong SAR, China; ^4^Department of Medicine, Queen Mary Hospital, The University of Hong Kong, Pok Fu Lam, Hong Kong SAR, China

**Keywords:** percutaneous coronary intervention, intracoronary imaging, intravascular ultrasound, optic coherence tomography, mortality, imaging paradox

## Abstract

**Background:**

There is a significant disparity between randomized controlled trials and observational studies with respect to any mortality benefit with intracoronary imaging during the percutaneous coronary intervention (PCI). This raises a suspicion that the imaging paradox, in which some operators may become over reliant on imaging and less proficient with angiography-guided PCI, might exist.

**Method:**

This was a retrospective cohort study from 14 hospitals under the Hospital Authority of Hong Kong between January 1, 2010 and December 31, 2017. Participants were patients who underwent first-ever PCI. The association between mortality risks of patients undergoing angiography-guided PCI and three tertiles (low, medium, and high) of the proportion of PCI done under intracoronary imaging guidance at a population level (background imaging rate), were evaluated after confounder adjustment by multivariable logistic regression.

**Results:**

In an adjusted analysis of 11,816 patients undergoing angiography-guided PCI, the risks of all-cause mortality for those were higher in the high-tertile group compared with the low-tertile group (OR, 1.45, 95% CI, 1.10–1.92, *P* = 0.008), the risks of cardiovascular mortality were higher in the high-tertile group compared with the low-tertile group (OR, 1.51, 95% CI, 1.08–2.13, *P* = 0.017). The results were consistent with multiple sensitivity analyses. Threshold analysis suggested that the mortality risks of angiography-guided PCI were increased when the proportion of imaging-guided PCI exceeded approximately 50%.

**Conclusions:**

The risks of the all-cause mortality and cardiovascular mortality were higher for patients undergoing angiography-guided PCI in practices with a higher background imaging rate.

## Background

Intracoronary imaging techniques (referred to as imaging hereafter), such as intravascular ultrasound (IVUS) and optical coherence tomography (OCT), can provide superior visualization than angiography alone in the assessment of lesion characteristics and poststenting results in percutaneous coronary intervention (PCI) (1–3). Randomized controlled trials (RCTs) have shown that imaging can reduce the rate of target vessel revascularization and myocardial infarction in selected patients, but the mortality outcomes are consistently neutral (4–8). In the contrary, many observational studies showed a mortality benefit with IVUS-guided PCI after confounder adjustment (9–11). In a meta-analysis of 31 RCTs and adjusted observational studies, IVUS was associated with lower mortality, but such association was neutralized if the analysis was restricted to RCTs (12). Although this disparity can be because of the unmeasured bias inherent to observational studies, it can also be contributed by operators' differential competency in performing angiography- and imaging-guided PCI. In practices with a high proportion of imaging-guided PCI, operators may become reliant on imaging and become less familiar with performing PCI with angiography alone. It is possible that the mortality benefit seen in observation studies is a reflection of worse survivals with angiography-guided PCI in operators who heavily rely on imaging guidance, and therefore not reproduced in RCTs which operators, by design, perform half of the interventions with angiography guidance.

The hypothesis that imaging may improve outcomes at an individual level, but paradoxically worsen outcomes for the patients receiving angiography-guided PCI with a high proportion of imaging use at a population level can be alarming. Thus, we aimed to determine the association between the utilization rate of imaging at a population level (referred to as “background imaging rate” hereafter) and mortality in patients receiving angiography-guided PCI.

## Methods

### Study Population and Design

Data from all patients who underwent first-ever PCI between January 1, 2010 and December 31, 2017 from all 14 public hospitals that performed PCI and recorded in a territorial-wide PCI registry were reviewed. Patients' baseline characteristics, exposures, and outcomes were retrieved from the PCI Registry and Clinical Data and Analysis Reporting System. The study was approved by the Institutional Review Board of the University of Hong Kong/Hospital Authority.

We included all adult patients (18 years of age or older) who underwent first-ever PCI and entered in the registry. Patients with prior PCI were excluded since both American and European guidelines have a class IIa recommendation for IVUS in the assessment of stent failure (13, 14).

### Definitions of Exposure and Outcome Variables

Imaging-guided PCI was defined as any utilization of IVUS or OCT throughout the procedure, and the remainder was defined as angiography-guided PCI. The proportion of imaging-guided PCI was calculated by dividing the number of imaging-guided PCI by the total number of PCI for each institution over each 2-year period (2010–2011, 2012–2013, 2014–2015, and 2016–2017). This proportion was considered as the background imaging rate, and was used to stratify into three tertiles (low, medium, and high) of imaging proportion groups. The primary outcome was all-cause mortality at 1 year after PCI. The secondary outcome was cardiovascular mortality 1 year after PCI.

### Statistical Analysis

All the analyses were performed with the prespecified outcome and statistical methods. Unadjusted analyses were made using chi-square tests for categorical variables and Student's *t*-test or ANOVA for continuous variables. First, we analyzed the effects of imaging guidance on mortality at an individual level. Multivariable logistic regression was performed to control for potential confounders selected *a priori* based on data in the published literature and biological plausibility. Confounder adjusted in the model were sex, age, tobacco use, diabetes mellitus, hypertension, dyslipidemia, cerebrovascular disease, chronic obstructive pulmonary disease, peripheral vascular disease, history of malignancy, previous myocardial infarction, previous coronary artery bypass surgery, previous heart failure, atrial fibrillation or flutter, anemia (hemoglobin < 13 g/dl for men, < 12 g/dl for women), estimated glomerular filtration rate < 30 ml/min/m^2^, indication for PCI [stable CAD, unstable angina, non-ST elevation myocardial infarction (NSTEMI), ST elevation myocardial infarction (STEMI)], PCI urgency (elective, urgent, emergency), number of affected epicardial artery, worst lesion characteristic (types A, B, C) (15), exclusive radial arterial access (16), hemodynamic instability (defined as acute pulmonary edema, or cardiogenic shock, or need for mechanical circulatory support, or ventricular tachycardia/fibrillation within 48 h before PCI), angiographic success, PCI period (2010–2011, 2012–2013, 2014–2015, and 2016–2017). Second, we analyzed the effects of background imaging rate on mortality restricted to patients receiving angiography-guided PCI, using the same multivariable logistic regression model.

### Sensitivity Analyses

In the first sensitivity analysis, we reclassified the background imaging rate into three groups according to an absolute percentage (<33%, 33–66%, and >66%) and repeated the analysis using the same regression model. Second, we reclassified the background imaging rate by hospital alone without dividing it into time periods, and repeat the analysis. Third, to exclude any selection bias such that patients with ultra-high risks or perceived poor survival were omitted from imaging, we repeated the analysis after the exclusion of patients surviving <30 days after PCI. Forth, we selected patients who underwent angiography-guided PCI in the low and high-tertile group, and constructed a propensity score model using the same variable used in the primary model. The analysis was repeated using inverse probability weighting based on the propensity score was used to balance for confounders.

The complete case method was adopted to address missing data in the primary statistical analysis. To test the robustness of our results, the multivariable logistic regression analysis was repeated with the entire cohort using the technique of multiple imputations by chained equations.

### Exploratory Analysis

We treated the background imaging rate as a continuous variable, and its association with mortality was evaluated using the same regression model. Furthermore, to identify a threshold which the background imaging rate was associated with mortality, we plotted the predicted risk of death at 1 year derived from the same model against the background imaging rate, fitting an M-spline curve with four interior knots.

Furthermore, we studied the effect modification on the relationship between background imaging rate and mortality by sex, PCI urgency, and PCI period (2010–2013 vs. 2014–2017), and introduced interaction terms to the logistic regression model.

Data management and statistical analyses were performed in Stata software, version 16 (StataCorp LP). A two-tailed *P* value of <0.05 was considered statistically significant.

## Results

### Patients and Characteristics

**Figure 1:**
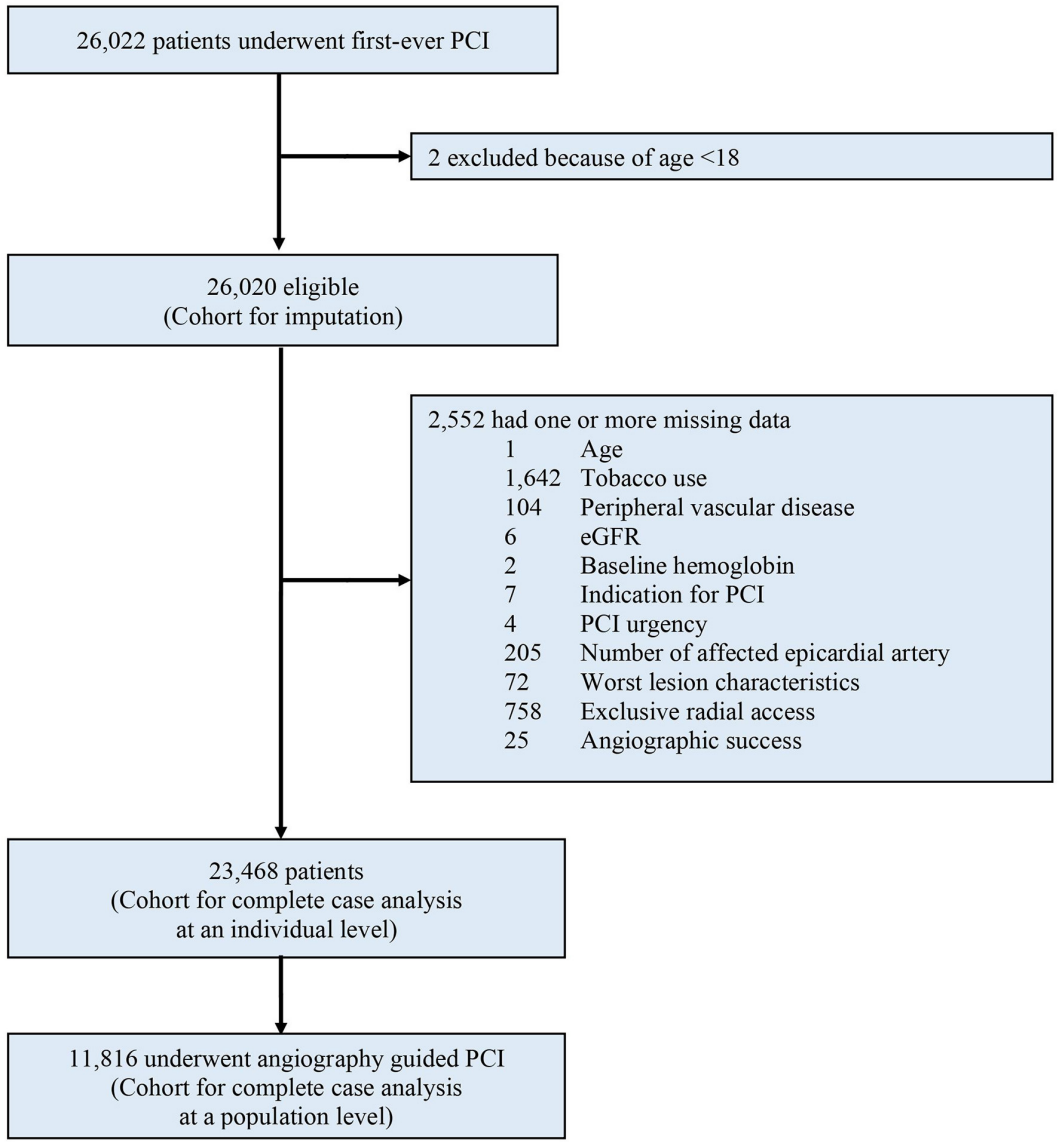
Study profile. PCI, percutaneous coronary intervention; eGFR, estimated glomerular filtration rate.

Between January 2010 and December 2017, a total of 26,022 patients were considered for inclusion: 2 (0.01%) were excluded due to age < 18. Of the remaining 26,020 patients analyzed, a total of 2,552 (9.8%) were excluded from the complete case analysis due to missing values in any of the variables used in the multivariable logistic regression model (Figure 1). Table 1 shows the baseline characteristics of the study population stratified by imaging guidance at an individual level. Table 2 shows the baseline characteristics of the patients undergoing angiography-guided PCI stratified by the background imaging rate. Table 3 shows the medication prescription on hospital discharge.

**Table 1:** Baseline characteristics of the study population were stratified by imaging guidance at an individual level.

**Characteristics**	**Imaging guided**	**Angiography guided**	***P* value**
N	12,932	13,088	
Female sex	2,953 (22.8%)	3,004 (23.0%)	0.82
Age, mean (SD)	64.5 (11.2)	65.1 (11.7)	<0.001
Tobacco use	5,554/12,253 (45.3%)	5,711/12,125 (47.1%)	0.005
Diabetes mellitus	4,514 (34.9%)	4,566 (34.9%)	0.97
Hypertension	8,194 (63.4%)	8,399 (64.2%)	0.17
Dyslipidemia	8,296 (64.2%)	8,203 (62.7%)	0.014
Cerebrovascular disease	1,198 (9.3%)	1,313 (10.0%)	0.036
Chronic obstructive pulmonary disease	313 (2.4%)	307 (2.3%)	0.69
Peripheral vascular disease	174/12,900 (1.3%)	208/13,016 (1.6%)	0.096
History of malignancy	680 (5.3%)	676 (5.2%)	0.74
Previous myocardial infarction	1,552 (12.0%)	1,684 (12.9%)	0.034
Previous coronary artery bypass surgery	251 (1.9%)	193 (1.5%)	0.004
Previous heart failure	1,012 (7.8%)	1,034 (7.9%)	0.82
Atrial fibrillation or flutter	689 (5.3%)	687 (5.2%)	0.78
Anemia	4,044 (31.3%)	4,288/13,086 (32.8%)	0.010
eGFR <30 ml/min/m^2^	547 (4.2%)	603/13,082 (4.6%)	0.14
Indication for PCI			<0.001
Stable CAD	2,706/12,931 (20.9%)	2,198/13,082 (16.8%)	
Unstable angina	3,035/12,931 (23.5%)	2,368/13,082 (18.1%)	
NSTEMI	5,970/12,931 (46.2%)	5,910/13,082 (45.2%)	
STEMI	1,220/12,931 (9.4%)	2,606/13,082 (19.9%)	
PCI urgency			<0.001
Elective	7,598 (58.8%)	7,454/13,084 (57.0%)	
Urgent	4,119 (31.9%)	2,932/13,084 (22.4%)	
Emergency	1,215 (9.4%)	2,698/13,084 (20.6%)	
Number of affected epicardial artery			<0.001
One vessel disease	5,547/12,798 (43.3%)	5,998/13,017 (46.1%)	
Two vessel disease	4,438/12,798 (34.7%)	4,214/13,017 (32.4%)	
Three vessel disease	2,813/12,798 (22.0%)	2,805/13,017 (21.5%)	
Left main disease (Unprotected)	1,264 (9.8%)	561 (4.3%)	
Worst lesion characteristics			<0.001
Type A	1,102/12,907 (8.5%)	1,483/13,041 (11.4%)	
Type B	8,519/12,907 (66.0%)	8,556/13,041 (65.6%)	
Type C	3,286/12,907 (25.5%)	3,002/13,041 (23.0%)	
Exclusive radial access	7,593/12,375 (61.4%)	5,931/12,887 (46.0%)	<0.001
Hemodynamic instability	1,048 (8.1%)	1,554 (11.9%)	<0.001
Acute pulmonary edema	454 (3.5%)	664 (5.1%)	<0.001
Cardiogenic shock	287 (2.2%)	512 (3.9%)	<0.001
Mechanical circulatory support	210 (1.6%)	298 (2.3%)	<0.001
Ventricular tachycardia/fibrillation	306 (2.4%)	508 (3.9%)	<0.001
Angiographic success	12,745/12,923 (98.6%)	12,547/13,072 (96.0%)	<0.001
Intravascular imaging	12,932 (100%)	0 (0%)	<0.001
Intravascular ultrasound	9,543 (73.8%)	0 (0%)	
Optic coherence tomography	3,539 (27.4%)	0 (0%)	
PCI period			<0.001
2010–2011	1,521 (11.8%)	3,712 (28.4%)	
2012–2013	2,320 (17.9%)	2,905 (22.2%)	
2014–2015	3,545 (27.4%)	3,093 (23.6%)	
2016–2017	5,546 (42.9%)	3,378 (25.8%)	

*PCI, percutaneous coronary intervention; SD, standard deviation; eGFR, estimated glomerular filtration rate; CAD, coronary artery disease; NSTEMI, non-ST elevation myocardial infarction; STEMI, ST elevation myocardial infarction*.

**Table 2:** Baseline characteristics of patients undergoing angiography-guided PCI stratified by background imaging rate.

**Background imaging rate**	**Low tertile**	**Medium tertile**	**High tertile**	***P* value**
*N*	7,190	4,344	1,554	
Female sex	1,593 (22.2%)	1,067 (24.6%)	344 (22.1%)	0.009
Age, mean (SD)	64.9 (11.5)	65.7 (12.0)	64.6 (12.0)	<0.001
Tobacco use	3,221/6,754 (47.7%)	1,770/3,903 (45.3%)	720/1,468 (49.0%)	0.019
Diabetes mellitus	2,631 (36.6%)	1,421 (32.7%)	514 (33.1%)	<0.001
Hypertension	4,728 (65.8%)	2,747 (63.2%)	924 (59.5%)	<0.001
Dyslipidemia	4,734 (65.8%)	2,628 (60.5%)	841 (54.1%)	<0.001
Cerebrovascular disease	726 (10.1%)	435 (10.0%)	152 (9.8%)	0.93
Chronic obstructive pulmonary disease	171 (2.4%)	97 (2.2%)	39 (2.5%)	0.80
Peripheral vascular disease	133/7,155 (1.9%)	60/4,319 (1.4%)	15/1,542 (1.0%)	0.017
History of malignancy	352 (4.9%)	236 (5.4%)	88 (5.7%)	0.29
Previous myocardial infarction	1,052 (14.6%)	487 (11.2%)	145 (9.3%)	<0.001
Previous coronary artery bypass surgery	88 (1.2%)	64 (1.5%)	41 (2.6%)	<0.001
Previous heart failure	605 (8.4%)	319 (7.3%)	110 (7.1%)	0.052
Atrial fibrillation or flutter	369 (5.1%)	257 (5.9%)	61 (3.9%)	0.008
Anemia	2,457/7,198 (34.2%)	1,313 (30.2%)	518/1,553 (33.4%)	<0.001
eGFR <30 ml/min/m^2^	327/7,187 (4.5%)	219/4,341 (5.0%)	57 (3.7%)	0.080
Indication for PCI				<0.001
Stable CAD	1,203/1,784 (16.7%)	770 (17.7%)	225 (14.5%)	
Unstable angina	1,291/1,784 (18.0%)	783 (18.0%)	294 (18.9%)	
NSTEMI	3,540/1,784 (49.3%)	1,653 (38.1%)	717 (46.1%)	
STEMI	1,150/1,784 (16.0%)	1,138 (26.2%)	318 (20.5%)	
PCI urgency				<0.001
Elective	4,510/7,186 (62.8%)	2,221 (51.1%)	723 (46.5%)	
Urgent	1,475/7,186 (20.5%)	954 (22.0%)	503 (32.4%)	
Emergency	1,201/7,186 (16.7%)	1,169 (26.9%)	328 (21.1%)	
Number of affected epicardial artery				0.007
One vessel disease	3,282/7,141 (46.0%)	2,021/4,343 (46.5%)	695/1,533 (45.3%)	
Two vessel disease	2,380/7,141 (33.3%)	1,324/4,343 (30.5%)	510/1,533 (33.3%)	
Three vessel disease	1,479/7,141 (20.7%)	998/4,343 (23.0%)	328/1,533 (21.4%)	
Left main disease (unprotected)	278 (3.9%)	191 (4.4%)	92 (5.9%)	
Worst lesion characteristics				<0.001
Type A	775/7,149 (10.8%)	587/4,340 (13.5%)	121/1,552 (7.8%)	
Type B	4,934/7,149 (69.0%)	2,661/4,340 (61.3%)	961/1,552 (61.9%)	
Type C	1,440/7,149 (20.1%)	1,092/4,340 (25.2%)	470/1,552 (30.3%)	
Exclusive radial access	3,393/7,141 (47.5%)	1,759/4,289 (41.0%)	779/1,457 (53.5%)	<0.001
Hemodynamic instability	715 (9.9%)	649 (14.9%)	190 (12.2%)	<0.001
Acute pulmonary edema	347 (4.8%)	252 (5.8%)	65 (4.2%)	0.016
Cardiogenic shock	172 (2.4%)	272 (6.3%)	68 (4.4%)	<0.001
Mechanical circulatory support	127 (1.8%)	120 (2.8%)	51 (3.3%)	<0.001
Ventricular tachycardia/fibrillation	227 (3.2%)	212 (4.9%)	69 (4.4%)	<0.001
Angiographic success	6,960/7,174 (97.0%)	4,169 (96.0%)	1,418 (91.2%)	<0.001
PCI period				<0.001
2010–2011	2,966 (41.3%)	746 (17.2%)	0 (0.0%)	
2012–2013	1,395 (19.4%)	1,120 (25.8%)	390 (25.1%)	
2014–2015	1,653 (23.0%)	820 (18.9%)	620 (39.9%)	
2016–2017	1,176 (16.4%)	1,658 (38.2%)	544 (35.0%)	

*PCI, percutaneous coronary intervention; SD, standard deviation; eGFR, estimated glomerular filtration rate; CAD, coronary artery disease; NSTEMI, non-ST elevation myocardial infarction; STEMI, ST elevation myocardial infarction*.

**Table 3:** Medication prescription on hospital discharge.

	**All patients**			**Angiography guided PCI only**			
**Medication**	**Imaging guided**	**Angiography guided**	***P* value**	**Low tertile**	**Medium tertile**	**High tertile**	***P* value**
*N*	12,932	13,088		7,190	4,344	1,554	
Aspirin	12,685 (98.1%)	12,572 (96.1%)	<0.001	6,851 (95.3%)	4,191 (96.5%)	1,530 (98.5%)	<0.001
P2Y12 inhibitor	12,793 (98.9%)	12,797 (97.8%)	<0.001	7,116 (99.0%)	4,169 (96.0%)	1,512 (97.3%)	<0.001
Proton pump inhibitor	7,984 (61.7%)	7,917 (60.5%)	0.039	4,406 (61.3%)	2,810 (64.7%)	701 (45.1%)	<0.001
Statin	12,431 (96.1%)	12,307 (94.0%)	<0.001	6,736 (93.7%)	4,113 (94.7%)	1,458 (93.8%)	0.085
Angiotensin blockade	8,817 (68.2%)	8,945 (68.3%)	0.77	4,710 (65.5%)	3,106 (71.5%)	1,129 (72.7%)	<0.001
Beta-blocker	9,423 (72.9%)	9,627 (73.6%)	0.21	5,248 (73.0%)	3,187 (73.4%)	1,192 (76.7%)	0.010
Anti-coagulant	545 (4.2%)	435 (3.3%)	<0.001	176 (2.4%)	199 (4.6%)	60 (3.9%)	<0.001

### Outcomes With Imaging Use at an Individual Level

At an individual level, the primary outcome of all-cause mortality at 1 year, developed in 608 (4.70%) patients in the imaging-guided group and 872 (6.66%) patients in the angiography-guided group (Table 4; Supplementary Figure 1). In adjusted analysis, this risk was not significantly different (odds ratio [OR], 0.93, 95% CI, 0.81–1.07, *P* = 0.31). The secondary outcome, cardiovascular mortality at 1 year, developed in 328 (2.54%) patients in the imaging-guided group and 543 (4.15%) patients in the angiography-guided group. In the adjusted analysis, this risk was not significantly different (OR, 0.89, 95% CI, 0.75–1.06, *P* = 0.19).

**Table 4:** Primary and secondary outcomes stratified by imaging guidance.

**Imaging guidance**	**Unadjusted absolute risk (95% CI)**	**Adjusted odds ratio (95% CI)**	***P* value**
**Primary outcome—all-cause mortality**			
Angiography guided PCI	6.66% (0.62–7.09%)	Reference	
Imaging guided PCI	4.70% (4.33–5.07%)	0.93 (0.81–1.07)	0.31
**Secondary outcome—cardiovascular mortality**			
Angiography guided PCI	4.15% (3.81–4.50%)	Reference	
Imaging guided PCI	2.54% (2.27–2.81%)	0.89 (0.751.06)	0.19

### Outcomes of Angiography-Guided PCI With Different Background Imaging Rates

**Figure 2:**
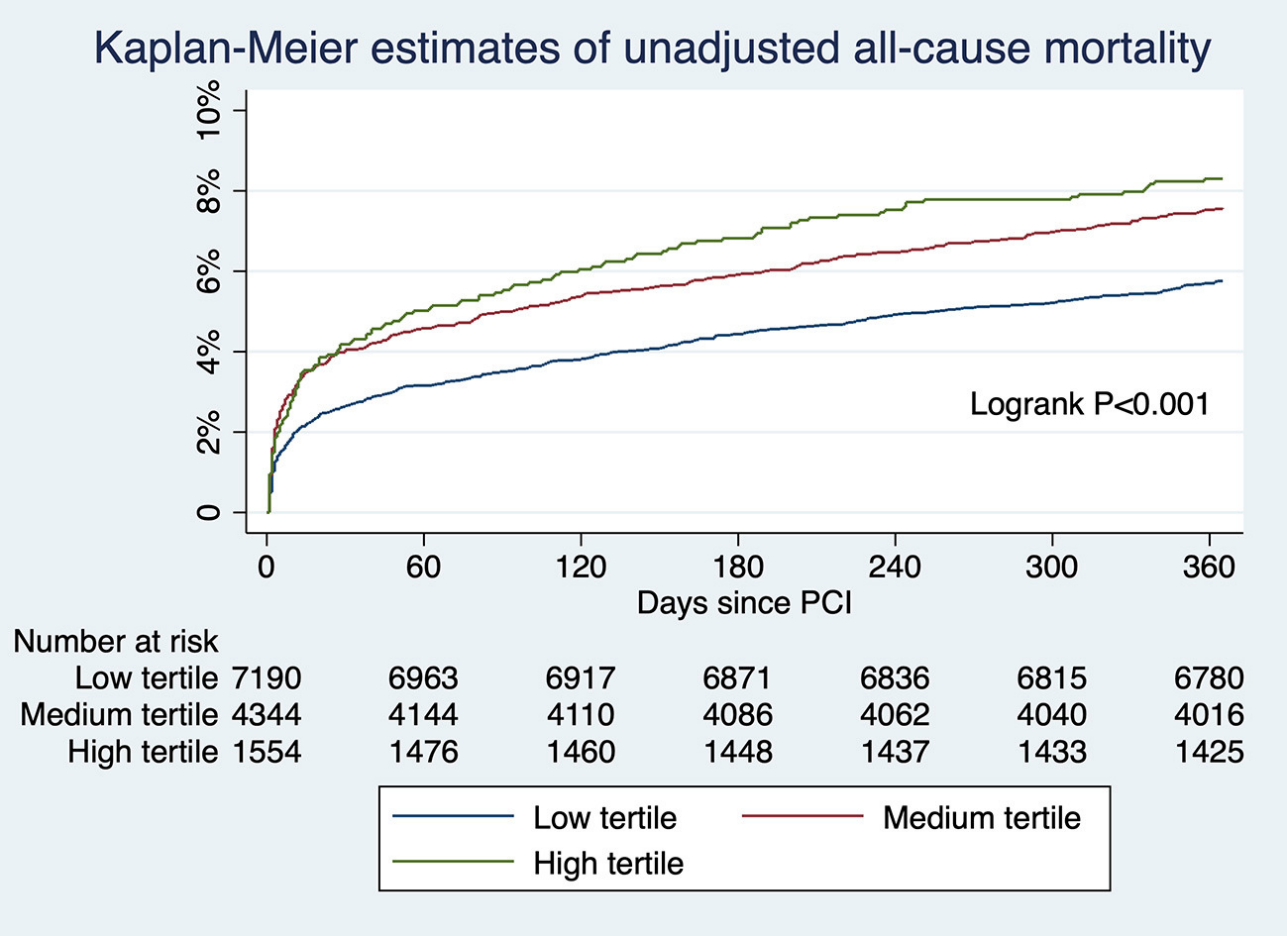
The unadjusted risks of all-cause mortality for those undergoing angiography-guided PCI were significantly different across tertiles of background imaging rate.

**Figure 3:**
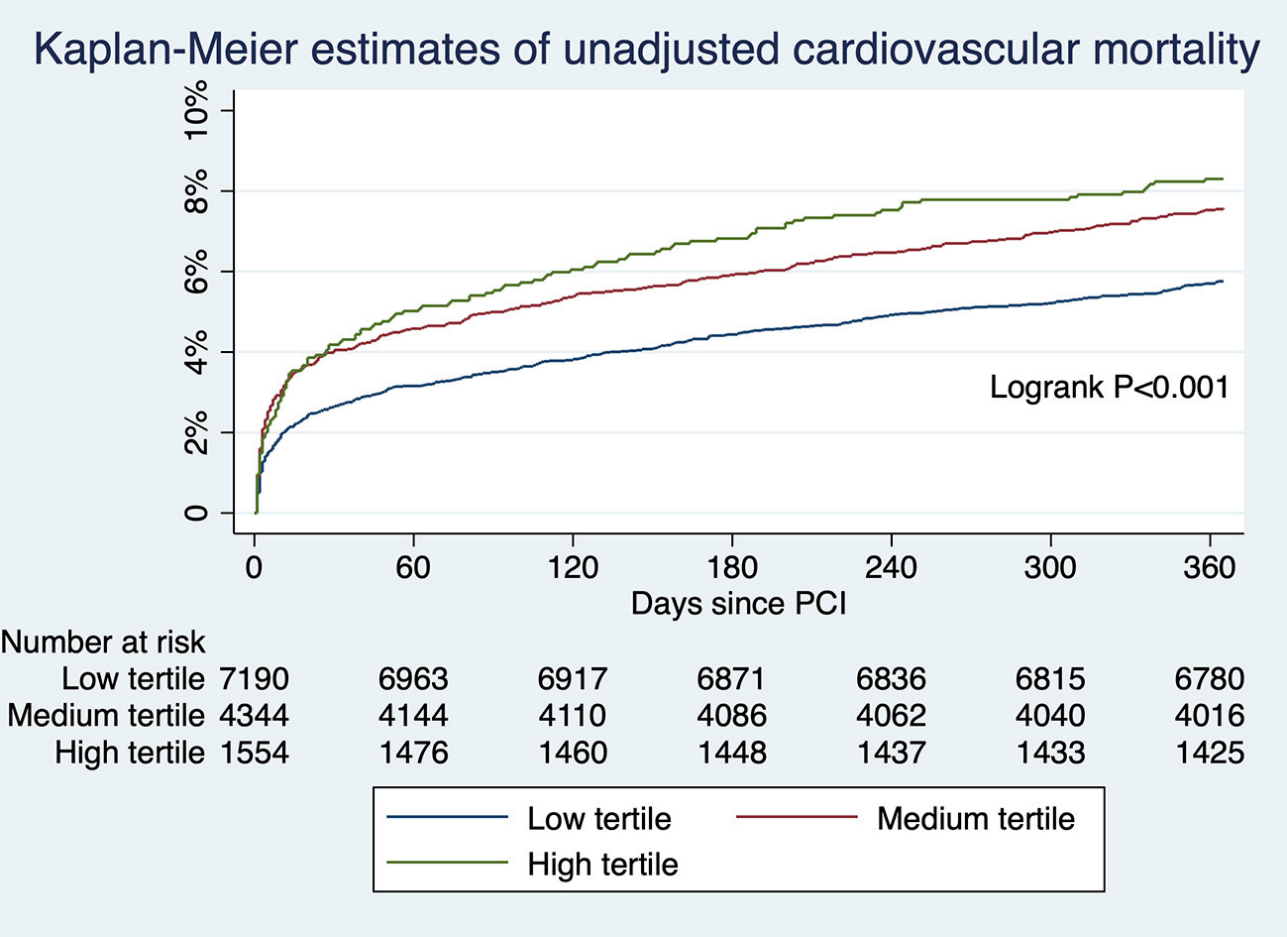
The unadjusted risks of cardiovascular mortality for those undergoing angiography-guided PCI were significantly different across tertiles of background imaging rate.

For patients undergoing angiography-guided PCI, the primary outcome developed in 414 (5.76%), 329 (7.57%), and 129 (8.30%) patients in the low, medium, and high tertile of background imaging rate, respectively (Figure 2; Table 5). In adjusted analysis, the risk was higher in the high-tertile group compared with the low-tertile group (OR, 1.45, 95% CI, 1.10–1.92, *P* = 0.008). The secondary outcome developed in 258 (3.59%), 202 (4.65%), and 83 (5.34%) patients in the low, medium, and high tertile, respectively (Figure 3; Table 5). In adjusted analysis, this risk was higher in the high-tertile group compared with the low-tertile group (OR, 1.51, 95% CI, 1.08–2.13, *P* = 0.017).

**Table 5:** Association between background imaging rate and outcomes restricted to the patient undergoing angiography-guided PCI.

**Background imaging rate**	**Unadjusted absolute risk (95% CI)**	**Adjusted odds ratio (95% CI)**	***P* value**
**Primary outcome—all-cause mortality**			
Low tertile	5.76% (5.21–6.30%)	Reference	
Medium tertile	7.57% (6.79–8.36%)	1.03 (0.84–1.25)	0.79
High tertile	8.30% (6.92–9.67%)	1.45 (1.10–1.92)	0.008
**Secondary outcome—cardiovascular mortality**			
Low tertile	3.59% (3.16–4.02%)	Reference	
Medium tertile	4.65% (4.02–5.28%)	0.89 (0.69–1.14)	0.35
High tertile	5.34% (4.22–6.46%)	1.51 (1.08–2.13)	0.017

### Sensitivity Analyses

After reclassification of background imaging rate according to an absolute percentage (low for <33%, medium for 33–66%, and high for >66%), the findings were consistent with the primary analysis: the risk of all-cause mortality was significantly higher in patients undergoing angiography-guided PCI in the high-proportion group (OR, 1.52; 95% CI, 1.11–2.10, *P* = 0.009) but not for those in the medium proportion group (OR, 1.16; 95% CI, 0.94–1.43, *P* = 0.15). Similarly, the risk of cardiovascular mortality was significantly higher in the high proportion (Supplementary Table 1 in the Supplementary Appendix). After reclassification of background-imaging rate according to hospital alone, the risks of all-cause mortality and cardiovascular mortality were significantly higher in both the high and medium proportion group (Supplementary Table 1 in the Supplementary Appendix).

After exclusion of patients surviving less than 30 days, the findings were consistent with the primary analysis: patients undergoing angiography-guided PCI in the high-tertile group had higher risks of all-cause mortality and cardiovascular mortality (Supplementary Table 1 in the Supplementary Appendix). After inverse probability-weighting based on the propensity score, the findings were also consistent with the primary analysis (Supplementary Table 1 in the Supplementary Appendix).

A total of 11 variables in the Cox regression model had missing data. After filling missing values with multiple imputations, patients undergoing angiography-guided PCI in the high-tertile group remained at higher risks of all-cause mortality (OR, 1.44; 95% CI, 1.12–1.85, *P* = 0.004) and cardiovascular mortality (OR, 1.46; 95% CI, 1.07–1.98, *P* = 0.016) compared with the low tertile, consistent with the primary analysis.

### Exploratory Analysis

**Figure 4:**
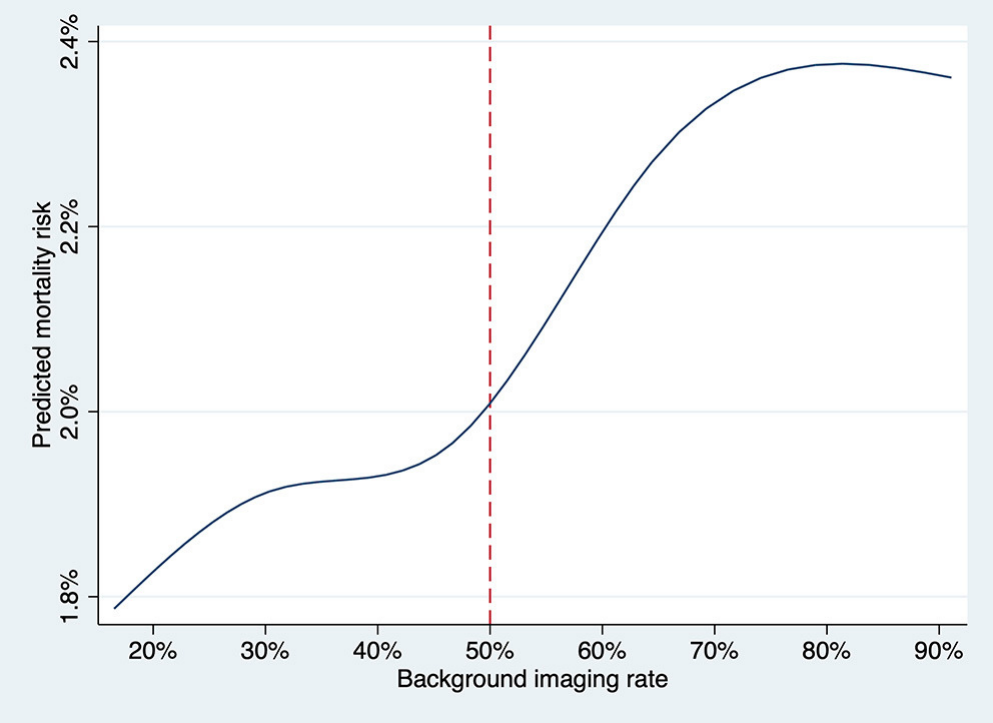
The predicted risk of death at 1 year for the patient undergoing angiography-guided PCI was plotted against the background imaging rate as a continuous variable, fitting an M-spline curve with four interior knots. The threshold for increased mortality with background imaging rate was ~50%.

Background imaging rate was treated as a continuous variable, and for each 10% absolute increase in the proportion, there was a significant increase risk of all-cause mortality (OR, 1.06; 95% CI, 1.01–1.11, *P* = 0.015) and an insignificant trend toward higher cardiovascular mortality (OR, 1.05; 95% CI, 0.99–1.12, *P* = 0.08) for patients undergoing angiography-guided PCI. The predicted risk of all-cause mortality for different levels of background imaging rate was shown in Figure 4. The threshold for increased mortality with background imaging rate was ~50%.

The background imaging rate-mortality relationship was not modified by sex (P-interaction [P-int] = 0.93 for medium tertile, P-int = 0.46 for high tertile), PCI urgency (P-int = 0.14 for medium tertile, P-int = 0.89 for high tertile) or PCI period (P-int = 0.77 for medium tertile, P-int = 0.88 for high tertile).

## Discussion

In this study, imaging-guided PCI was associated with a similar mortality rate at 1 year compared with angiography-guided PCI at an individual level. However, higher background imaging rate was associated with a paradoxically increase in all-cause mortality and cardiovascular mortality rates at 1 year for patients undergoing angiography-guided PCI. To our best knowledge, this relationship has not been previously described and we refer to it as the imaging paradox.

Many RCTs have shown that imaging could reduce major adverse cardiovascular events (MACEs), driven by a lower rate of target vessel revascularization but not mortality (4, 5, 7, 8). In the IVUS-XPL trial (n = 1,400), IVUS-guided PCI resulted in a lower risk of target lesion revascularization than angiography-guided PCI (4, 17). In the ULTIMATE trial (n = 1,448), IVUS reduced target lesion failure based on lesion-level analysis but not patient-level analysis (5). These findings are consistently discrepant with observational studies, where IVUS-guided PCI was associated with a reduction in MACEs including mortality (9–11). In a meta-analysis of 31 RCTs and adjusted observational studies, IVUS was associated with lower mortality, but such association was neutralized if the analysis was restricted to RCTs (12). This disparity may be contributed by operators' differential competency of performing angiography- and imaging-guided PCI. Since RCT are ethically bounded to be performed by operators competent in both angiography- and imaging-guided PCI, and by the process of randomization half of the PCI are done under angiography guidance, they may not reflect the real-world scenario in which operators are at different proficiency levels in imaging vs. angiography-guided PCI. The existence of the imaging paradox can potentially exaggerate the mortality benefits of imaging guidance seen in the observational studies.

The imaging paradox may originate from certain operators becoming over reliant on imaging guidance and less familiar with angiography-guided PCI. It is known that PCI outcomes are volume-dependent (18, 19). Operators who utilize imaging guidance routinely, will consequently have a lower volume of angiography-guided PCI. These operators may lose the ability to detect and interpret subtle details from angiograms. For example, it was shown that a careful read on angiography is adequate to guide stent optimization in calcified lesions (20, 21), nevertheless IVUS can provide superior sensitivity for detection of coronary calcification (22). Similarly, many other elements of PCI optimization, including landing zone, stent sizing, stent expansion, and apposition can be readily detectable by imaging (23–26). Operators who become overly reliant on imaging may become less sensitive to important angiographic manifestations, and resulting in suboptimal outcomes when performing angiography-guided PCI.

The imaging paradox has a resemblance to the radial paradox (27). It was noted in an RCT that clinical benefits of radial access were entirely confined to centers where the proportion of radial PCI was high (i.e., 80–98%) (28, 29), raising suspicion that results were merely a reflection of suboptimal outcomes with femoral access performed by operators who used radial access almost exclusively in pretrial procedures (30). We suspect that the imaging paradox is even more relevant than the radial paradox because (1) the visual superiority of imaging is far too superior to angiography-making operators easily become reliant (31), (2) there is no quick or easy way to improve angiography reading (32, 33), (3) the difference is in mortality.

Currently, imaging for PCI is considered adjunctive, and major American or European guidelines do not recommend for routine use (13, 14). Imaging is sometimes unsuitable for certain patients, and also requires additional procedural time, equipment, and cost (31, 34). Therefore, the ability to proficiently perform PCI under angiography guidance is still the backbone. While we recognize that the uptake of imaging is highly variable, with utilization rate of 30 to 80% in certain regions in Asia (35, 36). but only 5% or less in Europe and the USA (11, 37, 38), we believe that training programs should include solid and comprehensive training in angiography-guided PCI. Operators should perform a certain minimum volume of angiography-guided PCI to maintain proficiency and minimize complications. From our threshold analysis, we suspect that hospitals should maintain ~50% of the PCI performed under angiography guidance.

The imaging paradox has important implications in future design of RCT for imaging. The design should ensure that any benefit detected is truly attributable to imaging guidance. One method is to select centers and operators who have a good balance of angiography- and imaging-guided PCI during the pretrial period. Future studies should also not only focus on the effects of imaging on the individual level, but also on a population level. Our data can help generate hypothesis and aid sample size calculation for future RCTs.

This study had some limitations. First, the observational nature of the study conferred risks of unmeasured confounding and bias. It was possible that, for instance, general frailty or perceivable limited life expectancy could possibly incline operators to forgo imaging. However, we used multivariate analysis to minimize the effects of confounders, and the results were consistent in multiple sensitivity analysis including the exclusion of early non-survivors. More importantly, the existence of the imaging paradox cannot be studied in RCTs by nature. Second, analysis was not performed at the operator level, as the information available to us was incomplete, and PCI are commonly performed by multiple operators in this locality. However, institutional experience correlates more strongly with survivals than operator experience (39–41). Third, this study is not designed to detect a deskilling process. It is uncertain that whether operators highly skilled in angiography-guided PCI who transitioned to imaging-guided PCI might have been able to skilfully revert back to angiography-guided PCI when necessary, or, the imaging paradox is contributed by newly trained operators who predominantly perform imaging-guided PCI and struggle with angiography-guided PCI because of suboptimal training.

## Conclusion

In conclusion, we observed an increased mortality risk in the patients undergoing angiography-guided PCI in practices with a higher proportion of PCI done under intracoronary imaging guidance at a population level. The existence of the imaging paradox should call for appropriate training and maintenance of competency to improve outcomes for patients receiving angiography-guided PCI.

## Data Availability Statement

The original contributions presented in the study are included in the article/Supplementary Material, further inquiries can be directed to the corresponding author/s.

## Ethics Statement

The studies involving human participants were reviewed and approved by Institutional Review Board of the University of Hong Kong/Hospital Authority (Reference Number UW 20-176). Written informed consent for participation was not required for this study in accordance with the national legislation and the institutional requirements.

## Author Contributions

AN and C-WS were responsible for the conception and design of the study. AN analyzed the data collected by AI. AN interpreted the data. AN, PN, and L-TL drafted the manuscript. All authors revised and approved the final manuscript, and are accountable for the accuracy and integrity of the work.

## Conflict of Interest

The authors declare that the research was conducted in the absence of any commercial or financial relationships that could be construed as a potential conflict of interest.

## Publisher's Note

All claims expressed in this article are solely those of the authors and do not necessarily represent those of their affiliated organizations, or those of the publisher, the editors and the reviewers. Any product that may be evaluated in this article, or claim that may be made by its manufacturer, is not guaranteed or endorsed by the publisher.

## References

[1] Nishimura RA Edwards WD Warnes CA Reeder GS Holmes DR Tajik AJ. Intravascular ultrasound imaging: *in vitro* validation and pathologic correlation. J Am Coll Cardiol. (1990) 16:145–54. 10.1016/0735-1097(90)90472-2 2193046

[2] Yabushita H Bouma BE Houser SL Aretz HT Jang IK Schlendorf KH. Characterization of human atherosclerosis by optical coherence tomography. Circulation. (2002) 106:1640–5. 10.1161/01.CIR.0000029927.92825.F6 12270856

[3] Raber L Mintz GS Koskinas KC Johnson TW Holm NR Onuma Y. Clinical use of intracoronary imaging. Part 1: guidance and optimization of coronary interventions. An expert consensus document of the European Association of Percutaneous Cardiovascular Interventions. Eur Heart J. (2018) 39:3281–300. 10.1093/eurheartj/ehy285 29790954

[4] Hong SJ Kim BK Shin DH Nam CM Kim JS Ko YG. Effect of intravascular ultrasound-guided vs angiography-guided everolimus-eluting stent implantation: the IVUS-XPL randomized clinical trial. JAMA. (2015) 314:2155–63. 10.1001/jama.2015.15454 26556051

[5] Zhang J Gao X Kan J Ge Z Han L Lu S. Intravascular ultrasound versus angiography-guided drug-eluting stent implantation: the ULTIMATE trial. J Am Coll Cardiol. (2018) 72:3126–37. 10.1016/j.jacc.2018.09.013 30261237

[6] Kim BK Shin DH Hong MK Park HS Rha SW Mintz GS. Clinical impact of intravascular ultrasound-guided chronic total occlusion intervention with zotarolimus-eluting versus biolimus-eluting stent implantation: randomized study. Circ Cardiovasc Interv. (2015) 8:e002592. 10.1161/CIRCINTERVENTIONS.115.002592 26156151

[7] Bavishi C Sardar P Chatterjee S Khan AR Shah A Ather S. Intravascular ultrasound-guided vs angiography-guided drug-eluting stent implantation in complex coronary lesions: meta-analysis of randomized trials. Am Heart J. (2017) 185:26–34. 10.1016/j.ahj.2016.10.008 28267472

[8] Elgendy IY Mahmoud AN Elgendy AY Bavry AA. Outcomes with intravascular ultrasound-guided stent implantation: a meta-analysis of randomized trials in the era of drug-eluting stents. Circ Cardiovasc Interv. (2016) 9:e003700. 10.1161/CIRCINTERVENTIONS.116.003700 26980883

[9] Choi KH Song YB Lee JM Lee SY Park TK Yang JH. Impact of intravascular ultrasound-guided percutaneous coronary intervention on long-term clinical outcomes in patients undergoing complex procedures. JACC Cardiovasc Interv. (2019) 12:607–20. 10.1016/j.jcin.2019.01.227 30878474

[10] Hur SH Kang SJ Kim YH Ahn JM Park DW Lee SW. Impact of intravascular ultrasound-guided percutaneous coronary intervention on long-term clinical outcomes in a real world population. Catheter Cardiovasc Interv. (2013) 81:407–16. 10.1002/ccd.23279 21805605

[11] Mentias A Sarrazin MV Saad M Panaich S Kapadia S Horwitz PA. Long-Term outcomes of coronary stenting with and without use of intravascular ultrasound. JACC Cardiovasc Interv. (2020) 13:1880–90. 10.1016/j.jcin.2020.04.052 32819477 PMC7444477

[12] Buccheri S Franchina G Romano S Puglisi S Venuti G D'Arrigo P. Clinical outcomes following intravascular imaging-guided versus coronary angiography-guided percutaneous coronary intervention with stent implantation: a systematic review and bayesian network meta-analysis of 31 studies and 17,882 patients. JACC Cardiovasc Interv. (2017) 10:2488–98. 10.1016/j.jcin.2017.08.051 29153502

[13] Levine GN Bates ER Blankenship JC Bailey SR Bittl JA Cercek B. ACCF/AHA/SCAI guideline for percutaneous coronary intervention: a report of the American college of cardiology foundation/American heart association task force on practice guidelines and the society for cardiovascular angiography and interventions. Circulation. (2011). 124:e574–651. 10.1161/CIR.0b013e31823a5596 22064601

[14] Neumann FJ Sousa-Uva M Ahlsson A Alfonso F Banning AP Benedetto U. (2018). ESC/EACTS Guidelines on myocardial revascularization. Eur Heart J. (2019). 40:87–165. 10.1093/eurheartj/ehy855 30615155

[15] Ryan TJ Faxon DP Gunnar RM Kennedy JW King SB 3rd Loop FD. Guidelines for percutaneous transluminal coronary angioplasty. a report of the American college of cardiology/American heart association task force on assessment of diagnostic and therapeutic cardiovascular procedures (subcommittee on percutaneous transluminal coronary angioplasty). Circulation. (1988) 78:486–502. 10.1161/01.CIR.78.2.486 2969312

[16] Ferrante G Rao SV Juni P Da Costa BR Reimers B Condorelli G. Radial versus femoral access for coronary interventions across the entire spectrum of patients with coronary artery disease: a meta-analysis of randomized trials. JACC Cardiovasc Interv. (2016) 9:1419–34. 10.1016/j.jcin.2016.04.014 27372195

[17] Hong SJ Mintz GS Ahn CM Kim JS Kim BK Ko YG. Effect of intravascular ultrasound-guided drug-eluting stent implantation: 5-year follow-up of the IVUS-XPL randomized trial. JACC Cardiovasc Interv. (2020) 13:62–71. 10.1016/j.jcin.2019.09.033 31918944

[18] Fanaroff AC Zakroysky P Dai D Wojdyla D Sherwood MW Roe MT. Outcomes of pci in relation to procedural characteristics and operator volumes in the United States. J Am Coll Cardiol. (2017) 69:2913–24. 10.1016/j.jacc.2017.04.032 28619191 PMC5784411

[19] Fanaroff AC Zakroysky P Wojdyla D Kaltenbach LA Sherwood MW Roe MT. Relationship between operator volume and long-term outcomes after percutaneous coronary intervention. Circulation. (2019) 139:458–72. 10.1161/CIRCULATIONAHA.117.033325 30586696 PMC6340715

[20] Tuzcu EM Berkalp B De Franco AC Ellis SG Goormastic M Whitlow PL. The dilemma of diagnosing coronary calcification: angiography versus intravascular ultrasound. J Am Coll Cardiol. (1996) 27:832–8. 10.1016/0735-1097(95)00537-4 8613611

[21] Wang X Matsumura M Mintz GS Lee T Zhang W Cao Y. *In Vivo* calcium detection by comparing optical coherence tomography, intravascular ultrasound, and angiography. JACC Cardiovasc Imaging. (2017) 10:869–79. 10.1016/j.jcmg.2017.05.014 28797408

[22] Mintz GS Popma JJ Pichard AD Kent KM Satler LF Chuang YC. Patterns of calcification in coronary artery disease. a statistical analysis of intravascular ultrasound and coronary angiography in 1155 lesions. Circulation. (1995) 91:1959–65. 10.1161/01.CIR.91.7.1959 7895353

[23] Prati F Romagnoli E Burzotta F Limbruno U Gatto L La Manna A. Clinical impact of OCT findings during PCI: The CLI-OPCI II Study. JACC Cardiovasc Imaging. (2015) 8:1297–305. 10.1016/j.jcmg.2015.08.013 26563859

[24] Ino Y Kubo T Matsuo Y Yamaguchi T Shiono Y Shimamura K. Optical Coherence tomography predictors for edge restenosis after everolimus-eluting stent implantation. Circ Cardiovasc Interv. (2016) 9:10. 10.1161/CIRCINTERVENTIONS.116.004231 27688261

[25] Kang SJ Cho YR Park GM Ahn JM Kim WJ Lee JY. Intravascular ultrasound predictors for edge restenosis after newer generation drug-eluting stent implantation. Am J Cardiol. (2013) 111:1408–14. 10.1016/j.amjcard.2013.01.288 23433757

[26] Kobayashi N Mintz GS Witzenbichler B Metzger DC Rinaldi MJ Duffy PL. Prevalence, features, and prognostic importance of edge dissection after drug-eluting stent implantation: an ADAPT-DES intravascular ultrasound substudy. Circ Cardiovasc Interv. (2016) 9:e003553. 10.1161/CIRCINTERVENTIONS.115.003553 27402854

[27] Azzalini L Tosin K Chabot-Blanchet M Avram R Ly HQ Gaudet B. The benefits conferred by radial access for cardiac catheterization are offset by a paradoxical increase in the rate of vascular access site complications with femoral access: the campeau radial paradox. JACC Cardiovasc Interv. (2015) 8:1854–64. 10.1016/j.jcin.2015.07.029 26604063

[28] Le May MR Singh K Wells GA. Efficacy of radial versus femoral access in the acute coronary syndrome: is it the operator or the operation that matters? JACC Cardiovasc Interv. (2015) 8:1405–9. 10.1016/j.jcin.2015.06.016 26404191

[29] Valgimigli M Gagnor A Calabro P Frigoli E Leonardi S Zaro T. Radial versus femoral access in patients with acute coronary syndromes undergoing invasive management: a randomised multicentre trial. Lancet. (2015) 385:2465–76. 10.1016/S0140-6736(15)60292-6 25791214

[30] Le May MR Wells GA. Unraveling the radial paradox. Circ Cardiovasc Interv. (2017) 10:e004865. 10.1161/CIRCINTERVENTIONS.117.004865 28196899

[31] Koskinas KC Nakamura M Raber L Colleran R Kadota K Capodanno D. Current use of intracoronary imaging in interventional practice- results of a european association of percutaneous cardiovascular interventions (EAPCI) and japanese association of cardiovascular interventions and therapeutics (CVIT) clinical practice survey. Circ J. (2018) 82:1360–8. 10.1253/circj.CJ-17-1144 29540631

[32] Seto AH Abu-Fadel MS Sparling JM Zacharias SJ Daly TS Harrison AT. Real-time ultrasound guidance facilitates femoral arterial access and reduces vascular complications: FAUST (Femoral Arterial Access With Ultrasound Trial). JACC Cardiovasc Interv. (2010) 3:751–8. 10.1016/j.jcin.2010.04.015 20650437

[33] Farooq V Goedhart D Ludman P de Belder MA Harcombe A El-Omar M. Relationship between femoral vascular closure devices and short-term mortality from 271 845 percutaneous coronary intervention procedures performed in the United Kingdom between 2006 and 2011: a propensity score-corrected analysis from the British cardiovascular intervention society. Circ Cardiovasc Interv. (2016) 9(6). 10.1161/CIRCINTERVENTIONS.116.003560 27225421

[34] Alberti A Giudice P Gelera A Stefanini L Priest V Simmonds M. Understanding the economic impact of intravascular ultrasound (IVUS). Eur J Health Econ. (2016) 17:185–93. 10.1007/s10198-015-0670-4 25669755

[35] Jang JS Han KR Moon KW Jeon DW Shin DH Kim JS. The current status of percutaneous coronary intervention in korea: based on year (2014). cohort of Korean Percutaneous Coronary Intervention (K-PCI) registry. Korean Circ J. (2017) 47:328–40. 10.4070/kcj.2017.0071 28567083 PMC5449527

[36] Kodaira M Kuno T Numasawa Y Ohki T Nakamura I Ueda I. Differences of in-hospital outcomes within patients undergoing percutaneous coronary intervention at institutions with high versus low procedural volume: a report from the Japanese multicentre percutaneous coronary intervention registry. Open Heart. (2018) 5:e000781. 10.1136/openhrt-2018-000781 30018774 PMC6045738

[37] Moschovitis A Cook S Meier B. Percutaneous coronary interventions in Europe in (2006). EuroIntervention. (2010) 6:189–94. 10.4244/EIJV6I2A31 20562067

[38] Smilowitz NR Mohananey D Razzouk L Weisz G Slater JN. Impact and trends of intravascular imaging in diagnostic coronary angiography and percutaneous coronary intervention in inpatients in the United States. Catheter Cardiovasc Interv. (2018) 92:E410–E5. 10.1002/ccd.27673 30019831 PMC6258336

[39] Aikawa T Yamaji K Nagai T Kohsaka S Kamiya K Omote K. Procedural volume and outcomes after percutaneous coronary intervention for unprotected left main coronary artery disease-report from the national clinical data (J-PCI Registry). J Am Heart Assoc. (2020) 9:e015404. 10.1161/JAHA.119.015404 32347146 PMC7428587

[40] Hannan EL Wu C Walford G King SB 3rd Holmes DR Ambrose JA. Volume-outcome relationships for percutaneous coronary interventions in the stent era. Circulation. (2005) 112:1171–9. 10.1161/CIRCULATIONAHA.104.528455 16103238

[41] Lee JH Eom SY Kim U Lee CH Son JW Jeon DW. Effect of operator volume on in-hospital outcomes following primary percutaneous coronary intervention for st-elevation myocardial infarction: based on the 2014. Cohort of Korean Percutaneous Coronary Intervention (K-PCI) Registry. Korean Circ J. (2020) 50:133–44. 10.4070/kcj.2019.0206 31845555 PMC6974659

