# Supplementary material for: Survivals of Angiography-Guided Percutaneous Coronary Intervention and Proportion of Intracoronary Imaging at Population Level: The Imaging Paradox
Source: Front Cardiovasc Med. 2022 Feb 24;9:792837. doi: 10.3389/fcvm.2022.792837 (PMC8907484; doi:10.3389/fcvm.2022.792837)
Supplement: Supplementary file 1 [file Data_Sheet_1.docx]

**Supplementary Appendix**

**ENDPOINT DEFINITIONS**

**Death**

Deaths is classified as cardiovascular or non-cardiovascular. The cause of death will be determined by the principal condition that resulted in the death, not the immediate mode of death. Managing physicians will utilize all available information provided, along with clinical expertise, in their adjudication of the cause of death.

**Cardiovascular death**

Death due to cardiovascular causes. They include:

- death from acute myocardial infarction and its complications (e.g., arrhythmia, sudden arrest, heart failure)
- sudden cardiac death
- death from heart failure
- death from stroke
- death caused by complications of cardiovascular procedures
- death from cardiovascular hemorrhage (e.g., intracranial hemorrhage, non-procedural or non-traumatic vascular rupture (e.g., aortic aneurysm), or hemorrhage causing cardiac tamponade)
- death from other cardiovascular causes not included in the above categories but with a specific, known cardiovascular cause (e.g., pulmonary embolus or peripheral arterial disease)

**BASELINE VARIABLES DEFINITIONS**

**Estimated glomerular filtration rate**

Estimated glomerular filtration rate (eGFR) is calculated based on MDRD equation, expressed as:

186 x (Creatinine/88.4) - 1.154 x (Age) - 0.203 x (0.742 if female) x (1.210 if black)

where Creatinine is expressed in μmol/L.

**Anemia**

Anemia is defined as hemoglobin <13g/dL for men and hemoglobin <12g/dL for women.

**PCI urgency**

- Elective: Patient cardiac status has been stable in the days or weeks before the operation. The procedure can be deferred without increased risk of compromised cardiac outcome.
- Urgent: Procedure required during the same hospitalization to minimize chances of clinical deterioration or adverse outcome. Clinical conditions include (but are not limited to) acute or worsening chest pain, acute or worsening HF, acute MI, critical coronary stenosis, IABP support, UA with intravenous nitroglycerin, and rest angina.
- Emergency: Procedure required because of ongoing, refractory (difficult, complicated, and/or unmanageable), unrelenting cardiac compromise, with or without hemodynamic instability, and not responsive to any form of therapy except PCI.

**Figures**

**Figure S1: Unadjusted risks of all-cause mortality of individual patients stratified by imaging versus angiography guided percutaneous coronary intervention.**

**
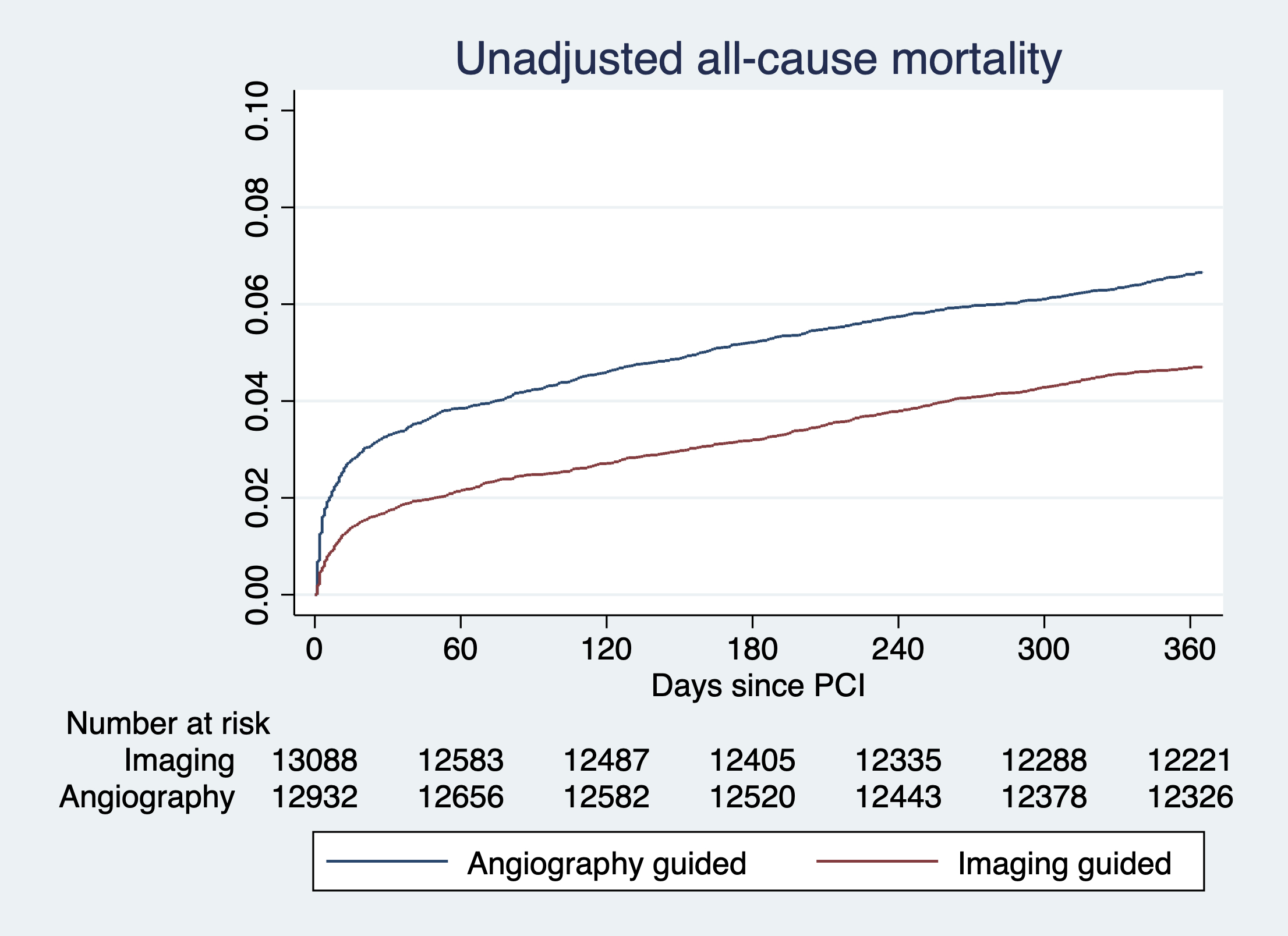
**

**Figure S2: Unadjusted risks of cardiovascular mortality of individual patients stratified by imaging versus angiography guided percutaneous coronary intervention.**

**
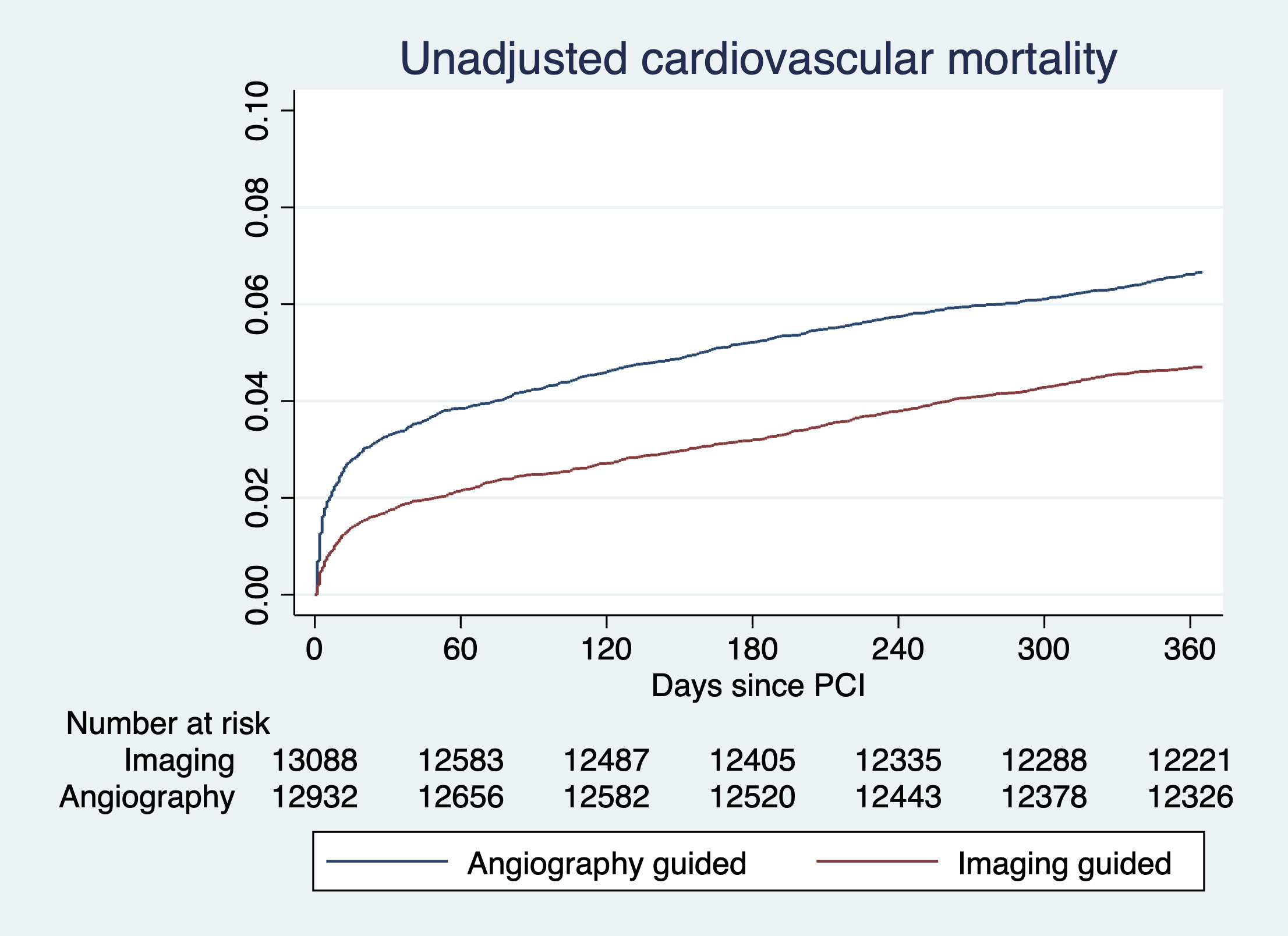
**

**Tables**

**Table S1: Results of sensitivity analysis. All analysis shown was restricted to patients undergoing angiography guided PCI.**

| Patient groups | All-cause mortality |  | Cardiovascular mortality |  |
| --- | --- | --- | --- | --- |
|  | Odds ratio (95% CI) | P value | Odds ratio (95% CI) | P value |
| Absolute background imaging rate |  |  |  |  |
| <33% | Reference |  | Reference |  |
| 33-66% | 1.16 (0.94-1.43) | 0.15 | 1.08 (0.83-1.40) | 0.59 |
| >66% | 1.52 (1.11-2.10) | 0.009 | 1.78 (1.21-2.61) | 0.003 |
|  |  |  |  |  |
| Background imaging rate by hospital alone |  |  |  |  |
| Low tertile | Reference |  | Reference |  |
| Medium tertile | 1.34 (1.11-1.62) | 0.002 | 1.46 (1.16-1.85) | 0.001 |
| High tertile | 1.56 (1.16-2.10) | 0.003 | 1.57 (1.07-2.32) | 0.021 |
|  |  |  |  |  |
| After exclusion of patients surviving <30 days |  |  |  |  |
| Low tertile | Reference |  | Reference |  |
| Medium tertile | 1.24 (0.97-1.60) | 0.09 | 1.19 (0.82-1.74) | 0.359 |
| High tertile | 1.66 (1.17-2.37) | 0.005 | 1.74 (1.03-2.94) | 0.039 |
|  |  |  |  |  |
| After inverse probability weighting of propensity score |  |  |  |  |
| Low tertile | Reference |  | Reference |  |
| Medium tertile | 1.27 (1.13-1.43) | <0.001 | 1.30 (1.12-1.51) | <0.001 |
| High tertile | 1.29 (1.10-1.51) | 0.002 | 1.45 (1.19-1.76) | <0.001 |
